# New Insights on the Inflammatory Role of *Lutzomyia longipalpis* Saliva in Leishmaniasis

**DOI:** 10.1155/2012/643029

**Published:** 2012-02-12

**Authors:** Deboraci Brito Prates, Théo Araújo-Santos, Cláudia Brodskyn, Manoel Barral-Netto, Aldina Barral, Valéria Matos Borges

**Affiliations:** ^1^Departamento de Biomorfologia, Instituto de Ciências da Saúde, Universidade Federal da Bahia, Avenida Reitor Miguel Calmon S/N, 40110-100 Salvador, BA, Brazil; ^2^Centro de Pesquisa Gonçalo Moniz (CPqGM), Fundação Oswaldo Cruz (FIOCRUZ), Rua Waldemar Falcão 121, 40296-710 Salvador, BA, Brazil; ^3^Faculdade de Medicina da Bahia, Universidade Federal da Bahia, Avenida Reitor Miguel Calmon S/N, 40110-100 Salvador, BA, Brazil; ^4^Instituto Nacional de Ciência e Tecnologia de Investigação em Imunologia (iii-INCT), Avenida Dr.Enéas de Carvalho Aguiar 44, 05403-900, São Paulo, SP, Brazil

## Abstract

When an haematophagous sand fly vector insect bites a vertebrate host, it introduces its mouthparts into the skin and lacerates blood vessels, forming a hemorrhagic pool which constitutes an intricate environment of cell interactions. In this scenario, the initial performance of host, parasite, and vector “authors” will heavily influence the course of *Leishmania* infection. Recent advances in vector-parasite-host interaction have elucidated “co-authors” and “new roles” not yet described. We review here the stimulatory role of *Lutzomyia longipalpis* saliva leading to inflammation and try to connect them in an early context of *Leishmania* infection.

## 1. Introduction

Leishmaniasis remains a serious problem in public health, endemic in 88 countries on four continents, but most of the cases occur in underdeveloped or developing countries [1]. Visceral Leishmaniasis (VL) is a progressive infection with fatal outcome in the absence of treatment. Approximately 90% of the VL cases registered in the Americas occur in Brazil and are concentrated in the Northeast region. In the New World, *Lutzomyia longipalpis* is the principal vector of *Leishmania infantum chagasi*, the agent of American Visceral Leishmaniasis [2].

The causes related to development of distinct clinical manifestations in leishmaniasis are multifactorial and reflect the complexity at the vector-pathogen-host interface [3]. Protozoan parasites of the genus *Leishmania *are the causative agents of the disease and are transmitted to the mammalian hosts by the bite of female phlebotomine sand flies during blood repast. For blood meal obtainment, sand flies introduce their mouthparts into the skin, tearing tissues, lacerating capillaries, and creating haemorrhagic pools upon which they feed [4]. The presence of sand fly saliva in the blood pool, the environment where the parasite encounters host cells, influences the development and functions of several leukocytes. In recent years, the importance of the interaction between components of sand fly saliva and host immune mechanisms in regulating infectivity and disease progression has become clearer and suggests their consequences to disease outcome in leishmaniasis [5].

The aspects involved in immune response resulting in resistance or susceptibility widely depend on the first attempt of host's innate response to contain infection that may influence on the predominance of a pattern of future host's immune adaptive response against *Leishmania*. Many studies have been performed to understand the mechanisms leading to protection or exacerbation of the disease however; relatively few studies have investigated the role of the sand- fly-derived salivary compounds in the innate immunity. In this paper we integrate the influence of sand fly bite with current ideas regarding the role of early steps of host inflammatory response against *Leishmania*.

## 2. Sand Fly Saliva: A Rich Field of Study

Sand fly vectors display a rich source of salivary biological active components to acquire blood from vertebrate hosts, a task not easy due the haemostatic, inflammatory and immune responses resultant from the bite [6]. Thus, it is not unexpected that many scientists have progressively investigated several aspects of sand fly saliva, concerning its composition and the range of mammalian response to it.

Among the New World species of sand fly which are vectors of *Leishmania*, *L. longipalpis* and its salivary gland content are the best studied. One of the first components related to *L. longipalpis* salivary gland was maxadilan [7], the most potent vasodilator peptide known and one of the two phlebotomine salivary proteins more extensively studied. Maxadilan is recognized by causing typical erythema during the feeding of *L. longipalpis* [8]. Further, it was described that maxadilan is able to modulate the inflammatory response by inhibiting cytokines such as TNF-*α*, by inducing IL-6 production, and by stimulating hematopoiesis [9–11]. Charlab et al. (1999) reported nine full clones and two partial cDNA clones from salivary gland from *L. longipalpis *[12]. In that work, they reported for the first time a hyaluronidase activity from sand fly saliva, an activity not yet described on phlebotomine sand flies, helping the diffusion of other pharmacological substances through the skin matrix [13]. It was also described an apyrase activity on *L. longipalpis* saliva which hydrolyses ATP and ADP to AMP, functioning as a potent antiplatelet factor [12, 14]. Interestingly, a 5′-nucleotidase activity is also present in *L. longipalpis* saliva exert vasodilator and antiplatelet aggregation role by converting AMP to adenosine [12]. One of the most abundant protein found in the *L. longipalpis* saliva is the *Yellow*-related protein [12, 13, 15, 16]. Our group has demonstrated that this family of proteins are the most recognized in sera from children living in an endemic area of visceral Leishmaniasis in Brazil [17] and by normal volunteers exposed to laboratory-reared *L. longipalpis* bites [18]. Recently, Xu et al. (2011) described the structure and function of a *yellow* protein LJM 11 [19]. In this report, the authors described that yellow proteins from *L. longipalpis *saliva act as binder of proinflammatory biogenic amines such as serotonin, histamine, and catecholamines [19]. One member of the D7 family of proteins (commonly found in dipterans saliva) is present in *L. longipalpis* [12]. The exact function of this protein in sand fly saliva is still unknown. However, its role on mosquito's saliva suggests that it could act as anticoagulant or binding biogenic amines avoiding host inflammatory events [12, 15].

Herein, we present some of the most studied proteins related to *L. longipalpis* saliva. (See [6, 15, 16, 20] for more details about this topic). Although many of them have been associated with blood-feeding, their biological functions remain undefined. Nevertheless, by modulating the host haemostatic and inflammatory response, this yet unreported sand fly salivary content remains as a research challenge, acting on host immunity to *Leishmania* during transmission and establishment of infection.

## 3. Immune Response to *Lutzomyia longipalpis *Saliva against *Leishmania*

**Figure 1:**
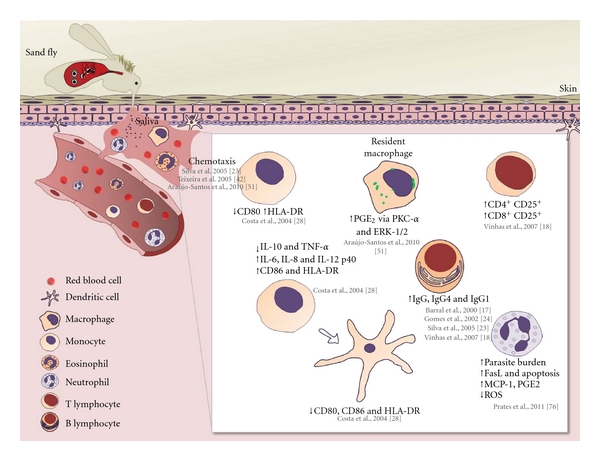
Roles of *Lutzomyia longipalpis* saliva in host immune response cell. After *L. longipalpis *saliva injection a set of events can be triggered in the host immune response. Herein, we summarized the roles of saliva on major cell populations involved in the host immune response against *Leishmania* infection.

There are several studies contributing to a better understanding of *L. longipalpis* saliva effects on host immunity to *Leishmania* infection. A brief exposition of these major contributions in the last 10 years is shown in Figure 1.

In mice, salivary products seem to exacerbate the infection with *Leishmania* and may, in fact, be mandatory for establishment of the parasite in vertebrate hosts. It has been shown that components of *L. longipalpis* or *Phlebotomus papatasi* salivary gland lysates mixed with *Leishmania major *resulted in substantially larger lesions compared to controls [21, 22]. Our group have shown that repeated exposure of BALB/c mice to *L. longipalpis *bites leads to local inflammatory cell infiltration comprised of neutrophils, macrophages and eosinophils [23]. Total IgG and IgG1 antibodies react predominantly with three major protein bands (45, 44, and 16 kD) from insect saliva by Western blot [23]. The injection of immune serum previously incubated with salivary gland homogenate induced an early infiltration with neutrophils and macrophages, suggesting the participation of immune complexes in triggering inflammation [23].

 We have shown that in endemic areas natural exposures to noninfected sand fly bites can influence the epidemiology of the disease [17, 24]. We observed that people who presented antibodies against saliva of *L. longipalpis* also showed DTH anti-*Leishmania,* suggesting that the immune response against saliva of the vector could contribute to the induction of a protective immune response against the parasite. Recently, in a prospective study this data was reinforced by Aquino et al. (2010) evaluating 1,080 children from 2 endemic areas for VL [25]. There was a simultaneous appearance of antibodies anti-saliva and an anti-*Leishmania* DTH, or a cellular response against the parasite [25], supporting the idea that eliciting immunity against saliva could benefit the induction of a protective response against the parasite. The anti-sand fly antibodies can serve as epidemiological marker of vector exposure in endemic areas. In fact, we demonstrated that two salivary proteins, called LJM 17 and LJM 11, were specifically recognized by humans exposed to *L. longipalpis*, but not* Lutzomyia intermedia* [26]. We also evaluated the specificity of anti*-L. longipalpis* in a panel of 1,077 serum samples and verified that LJM 17 and LJM 11 together in an ELISA assay identified the effectiveness of these proteins for the prediction of positivity against salivary gland sonicate (SGS) [27]. In experimental model using C57BL/6 mice, immunization with LJM 11 triggered DTH response and decrease the diseased burden after *L. major *infection [19].

 We also characterized the immunological patterns following sand fly saliva exposure, using healthy volunteers exposed to laboratory-reared *L. longipalpis* [18]. We noticed high levels of IgG1, IgG4, and IgE antibodies anti-saliva. Furthermore, following *in vitro* stimulation with salivary gland sonicate, there was an increased frequency of CD4(+)CD25(+) and CD8(+)CD25(+) T cells as well as IFN-*γ* and IL-10 synthesis. Strikingly, 1 year after the first exposure, PBMC from the volunteers displayed recall IFN-*γ* responses that correlated with a significant reduction in infection rates using a macrophage-lymphocyte autologous culture. Together, these data suggest that human immunization against sand fly saliva is feasible and recall responses are obtained even 1 year after exposure, opening perspectives for vaccination in man [18].

Sand fly saliva also seems to exert a direct effect on human antigen presenting cells.* L. longipalpis* SGS inhibited IL-10 and TNF-*α* production but induced IL-6, IL-8, and IL-12p40 production by LPS-stimulated monocytes and dendritic cells [28]. Besides cytokine production, sand fly saliva also interfered with the expression of costimulatory molecules in macrophages (reduced CD80 and increased HLA-DR expression) and in monocytes (increased CD80 and HLA-DR expression). During dendritic cell differentiation induced by CD40L, a slight reduction in CD80, CD86, HLA-DR, and CD1a expression were also observed [28].

Whereas enhancement of *Leishmania *transmission by saliva is probably due to immunomodulatory components of sand fly saliva, an explanation of the anti-*Leishmania *effect resulting from host immunization against salivary antigen is not straightforward. Immunity in this system could derive from neutralization of salivary immunomodulators such as the peptide maxadilan from *L. longipalpis* (as reviewed in [22]). Alternatively, immunity could derive from a DTH reaction at the site of the bite generated by a cellular response to salivary antigens injected by the fly [29, 30]. This particular reaction could turn the lesion and its surroundings into an inhospitable site for the establishment of *Leishmania* infection in the new host, or it could modify the environment priming the initial events of the host immune reaction to *Leishmania.*

The disease exacerbative properties of saliva, often resulting from the bioactive property of one or more of its molecules, should not be confounded with antigenic molecules in saliva that induce an adaptive immune response in the host. This acquired immunity can be either protective or exacerbative depending on the nature and dominance of the salivary components of a vector species. Exposure to uninfected bites of the sand fly *P. papatasi* induces a strong delayed-type hypersensitivity response and IFN-*γ* production at the bite site that confers protection in mice challenged by* L. major*-infected flies [29]. By contrast, acquired immunity to* L. intermedia* saliva results in disease exacerbation not protection [31]. Moreover, *P. papatasi* saliva, despite its overall protective property, contains molecules that alone induce a protective (PpSP15) or exacerbative (PpSP44) immune response in the host [32, 33]. It is likely that *L. intermedia* saliva also contains molecules with similar profiles despite the overall exacerbative effect of total saliva.

Recently, we developed a model for visceral Leishmaniasis (VL) in hamsters, using an intradermal inoculation in the ears of 100,000* L. chagasi *parasites together with *L. longipalpis* saliva to mimic natural transmission by sand flies [34]. Hamsters developed classical signs of VL rapidly, culminating in a fatal outcome 5-6 months postinfection. Immunization with 16 DNA plasmids coding for salivary proteins of *L. longipalpis* resulted in the identification of LJM19, a novel 11-kDa protein that protected hamsters against the fatal outcome of VL. LJM19-immunized hamsters maintained a low parasite load that correlated with an overall high IFN-*γ*/TGF-*β* ratio and inducible NOS expression in the spleen and liver up to 5 months post-infection. Importantly, a delayed-type hypersensitivity response with high expression of IFN-*γ* was also noted in the skin of LJM19-immunized hamsters 48 h after exposure to uninfected sand fly bites. Induction of IFN-*γ* at the site of bite could partly explain the protection observed in the viscera of LJM19-immunized hamsters through direct parasite killing and/or priming of anti*-Leishmania* immunity. Recently, Tavares et al. [35] showed that LJM19 was also able to protect hamsters against an infection composed by *Leishmania braziliensis *plus saliva of* L. intermedia*, the vector responsible for the transmission of this parasite in Brazil [35]. The immunization also induced a higher ratio of IFN-*γ*/TGF-*β* production in the cells from lymph nodes draining the infection site. Collin et al., (2009) immunized dogs using intradermal injections of DNA codifying salivary proteins of* L. longipalpis* (LJM17 and LJL 143), followed by injection of recombinant *Canarypox virus* containing the same genes [36]. They also observed a potential protective response against *Leishmania*, showing high concentrations of IFN-*γ* in PBMC stimulated with recombinant salivary proteins. Importantly, the bite of uninfected sand flies resulted in a strong DTH characterized by high amount of IFN-*γ* and low levels of TGF-*β* [36]. Together, these results point out the possibility to immunize against leishmaniasis using defined proteins of vector's saliva against *Leishmania*.

## 4. Early Steps of Host-Vector-*Leishmania* Interplay: Cell Recruitment Induced by Saliva

It is well established that the first steps in leishmaniasis are critical in determining the development of the disease. In order to understand this critical moment, several reports have investigated the early recruitment of cells induced by both *L. longipalpis* saliva alone or coinoculated with *L. chagasi*. Sand fly saliva is able to induce an inflammatory process in the host by recruiting different cells into the bite site. In fact, it was verified that *L. longipalpis* salivary gland lysate markedly modifies the inflammatory response to infection with *L. braziliensis* in BALB/c mice [37]. The saliva-associated lesions progressed to extensive accumulations of heavily parasitized epithelioid macrophages, with persistent neutrophilia and eosinophilia [37]. Eosinophilia has also been described in dogs intradermally inoculated with *L. longipalpis* saliva associated with *L. chagasi* promastigotes [38]. Interestingly, this inflammatory response was not observed in animals that received saliva or parasites alone [38]. The significance of this in the context of Leishmaniasis remains to be investigated. However, this phenomena is not exclusive to *L. longipalpis* saliva once eosinophils were described in the inflammatory course at the site of immunization of mice with the salivary recombinant 15-kDa protein from *P. papatasi*, the sand fly species vector of *Leishmania major* [32]. It is well established the abundant presence of eosinophils in both inflammatory site and allergic response. Activated eosinophils release lipid mediators as PAF, prostaglandins, leukotrienes, and lipoxins, as well as cytokines IL-10 and IL-8 that, in conjunct, trigger vasodilatation and leukocyte chemotaxis (reviewed in [39]). In the context of sand fly bite, this eosinophilic reaction could favor vector feeding but creates an unfriendly environment for *Leishmania* parasites.

Host cell infiltration induced by sand fly bite is the most physiologic approach to reinforce the inflammatory role of vector saliva. This event has been explored using *P. papatasi*, in which saliva-induced DTH response observed was associated to a possible fly adaptation to manipulate host immunity for the vector's own advantage [30]. Concerning *L. longipalpis* saliva, our group investigated the initial vertebrate reactions against sand fly saliva. We demonstrated that repeated exposures of BALB/c mice to *L. longipalpis* bites lead to an intense and diffuse inflammatory infiltrate characterized by neutrophils, eosinophils, and macrophages [23]. This response was observed by histological analysis of the ear dermis from exposed mice as early as 2 hours and was sustained up to 48 hours after challenge with the *L. longipalpis* salivary sonicate [23]. Moreover, the injection of immune serum previously incubated with salivary gland homogenate induced an early infiltration with neutrophils and macrophages, suggesting the participation of immune complexes in triggering inflammation [23]. An elegant and remarkable visual advance obtained by two-photon intravital imaging has recently demonstrated that the neutrophils represent the first cell population which is recruited to *Phlebotomus duboscqi* bite site [40]. Although the participation of vector salivary components had not been directly attributed to this inflammatory event by the authors, we could not discharge this possibility considering diverse data showing that saliva from different sand flies species exert chemotaxis. As neutrophils were observed on *L. longipalpis* bite site [23] the implications of its saliva on this cells will be further discussed in this paper.

In addition to *in vivo* models, cell chemotaxis induced by saliva has also been observed* in vitro*. This is of particular interest, indicating that *L. longipalpis* salivary components can act directly as inflammatory mediator. Using transwell system, Zer et al. (2001) showed the direct chemotatic effect of saliva on BALB/c peritoneal macrophages. In the same work, it was demonstrated that *L. longipalpis* saliva is able to both increase the percentage of macrophages that became infected with *Leishmania* in BALB/c and C3H/HeN mice and exacerbate the parasite load in these cells [41]. The authors discuss the possibility that, during natural transmission, saliva could reduce the promastigote exposure to the immune system by attracting host cells to the bite site and by accelerating the uptake of these parasites.

Exploring a straightforward and consistent model—the mouse air pouch—to investigate the inflammatory response induced by *L. longipalpis*, our group has described that *L. longipalpis* salivary gland sonicate was able to induce not only macrophages, but also neutrophil and eosinophil recruitment after 12 h in BALB/c [42]. The increased macrophage recruitment was linked to production of chemokine CCL2/MCP-1 and expression of its receptor CCR2 in the air pouch lining tissue. It was observed that *L. longipalpis* also synergizes with *L. chagasi* to recruit more inflammatory cells to the site of inoculation [42]. This is noteworthy because it increases the availability of “safe targets,” the macrophages, for parasite evasion of the effector immune responses [43]. Interestingly, the recruitment profile observed in BALB/c was not observed in C57BL/6 mice, indicating that the same salivary components can induce diverse inflammatory effects depending on the host background [42]. However, because of limited number of cells that can be recovered on the air pouch model, some questions concerning early inflammatory events could not be investigated. Alternatively, the peritoneal cavity has been employed to this kind of study allowing the collection of high number of immigrating cells [44, 45]. In this regard, leukocyte recruitment into peritoneal cavity induced by *L. longipalpis* saliva has been evaluated in both BALB/c and C57BL/6 mouse strains [45]. In this work, significant neutrophil recruitment was observed six hours after administration of saliva, *L. major*, or saliva plus *L. major*. However, in BALB/c mice, all stimuli were able to induce more neutrophil migration than in C57BL/6 mice. Seven days later, it was observed that all stimuli were able to induce higher numbers of eosinophils and mononuclear cells in BALB/c when compared with C57BL/6 mice [45]. This study focused on the effect of saliva from *L. longipalpis* on adaptive immunity, evaluating CD4+ T lymphocyte migration and production of IL-10 and IFN-*γ* cytokines [45].

### 4.1. Inflammatory Events Triggered by *L. longipalpis * Saliva

**Figure 2:**
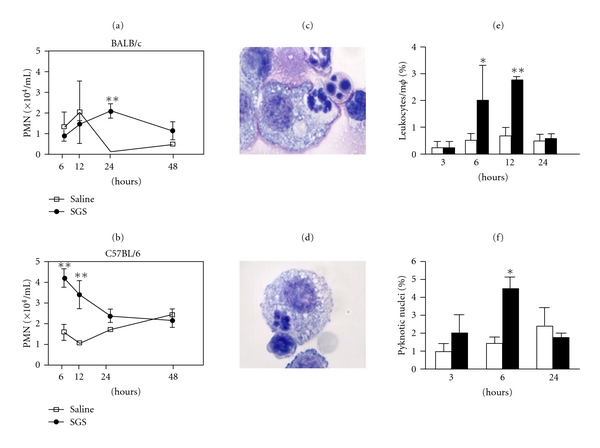
Neutrophil influx, apoptosis, and phagocytosis into BALB/c and C57BL/6 peritoneal cavity in response to *L. longipalpis* saliva. Mice were injected with endotoxin-free saline or *L. longipalpis* salivary gland sonicate (SGS) (0.5 pair/animal). After stimulation, peritoneal cavities were washed and differential cell counts were performed on Diff-Quik stained cytospin preparations. (a-b) Kinetics of neutrophil recruitment in BALB/c (a) and C57BL/6 (b) mice. (c-d) Representative events of C57BL/6 neutrophil phagocytosis by macrophages on Diff-Quik stained cytospin (magnification 1000x). (e-f) Phagocytosis of C57BL/6 leukocytes by macrophages (e) and neutrophil apoptosis (f) after stimulation with SGS (•) or saline (□). Data shown are from a single experiment representative of three independent experiments. Values represent means ± SEM of five mice per group. *P < 0.05 and **P < 0.01.

Neutrophils rapidly accumulate at the inflammatory site (as reviewed in [46]) and have been described on the sand fly bite site [23, 40]. Focusing on inflammatory events triggered by *L. longipalpis* saliva using the peritoneal model, we could observe a distinct kinetic of neutrophil recruitment to the peritoneal cavity of BALB/c and C57BL/6 mice (Figure 2). A late neutrophil influx was observed in BALB/c mice (Figure 2(a)), whereas in C57BL/6 mice neutrophils were already evident in the first hours after *L. longipalpis* saliva inoculation compared to mice injected with endotoxin-free saline (Figure 2(b)). 

The link between neutrophil recruitment induced by *L. longipalpis* saliva and other events which initiate and switch off the inflammatory response is an attractive field to be explored. Inflammation resolution is regulated by the release of mediators that contribute to an orchestrated sequence of events [47]. For simplicity, they result in predominance of neutrophils in the inflamed area which are later replaced by monocytes that differentiate into macrophages. During the resolution, inflammatory cells undergo apoptosis and are phagocytosed. Clearance of apoptotic cells by macrophages drives a response characterized by release of anti-inflammatory mediators [48]. Such safe removal of apoptotic cells has been implicated in exacerbation of *Leishmania* infection [49, 50]. The influence of *L. longipalpis* saliva in the time course of inflammation could be observed in cytospin preparations of the peritoneal cells from C57BL/6 mice. Neutrophils in contact with or phagocytosed by macrophages were observed at six hours (Figures 2(c) and 2(d)) and leukocyte phagocytosis by macrophages was an early event as well (Figure 2(e)). Moreover, apoptotic neutrophils were evident in C57BL/6 mice in the presence of saliva (Figure 2(f)). Therefore, components of sand fly saliva are able to both recruit and induce proapoptotic effects on neutrophils. These findings, in the scenario of anti-inflammatory clearance of apoptotic cells, add to the notion of beneficial effects of vector saliva on *Leishmania* transmission. Further work on mediators and mechanisms involved in this process is necessary.

## 5. Host Macrophage Response to * L. longipalpis* Saliva

Sand fly saliva displays an important role in the macrophage response by triggering the recruitment [42, 51] and suppressing the killing of parasites within macrophages [41, 52]. In this regard, *P. papatasi* saliva inhibits the NO production in macrophages treated with IFN-*γ* [52] and *L. longipalpis *saliva hampers *Leishmania *antigen presentation to T lymphocytes by macrophages [53] as well as upregulates the IL-10 production related with NO suppression in macrophages infected with *L. amazonensis* [54]. Moreover, pure adenosine from *P. papatasi *saliva decreases NO production in murine macrophages [55] and maxadilan peptide present in *L. longipalpis *saliva upregulates IL-6, IL-10, and TGF-*β* cytokine responses of LPS-activated macrophages and downregulates IL-12, TNF-*α*, and NO associated with *L. major *killing [56]. Despite this, few research reports cover the cellular pathways involved in sand fly saliva modulation of macrophage response. Previous study showed that maxadilan acts on PAC-1 receptor in LPS-activated macrophages and inhibits TNF-*α* production whereas it increases IL-6 and PGE_2_ [11], and the authors suggest the participation of cAMP activation by maxadilan in this process.

**Figure 3:**
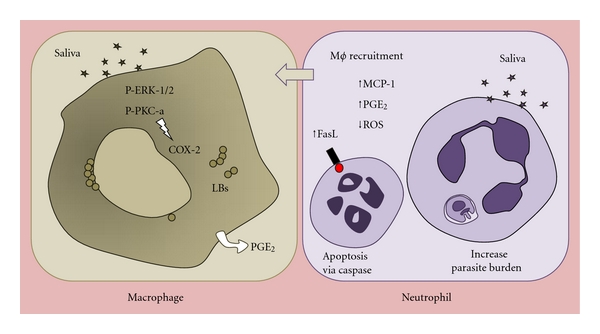
Effects of *Lutzomyia longipalpis *saliva on macrophage activation and neutrophil apoptosis. Macrophages and neutrophils are the first host cells to contact *Leishmania *after sand fly bite. Saliva triggers macrophages activation by lipid bodies formation committed with the PGE_2_ production via COX-2 after phosphorilation of kinases. On the other hand, saliva induces neutrophil apoptosis by caspase and FasL activation. In addition, neutrophils activated by saliva become susceptible to *Leishmania chagasi *and release MCP-1, which is associated with macrophage recruitment. This scenario promoted by *L. longipalpis *saliva can contribute to *Leishmania* transmission in the early times of infection.

Although the literature abounds with reports on the effects of sand fly saliva in the immune response and infection, the effect of whole sand fly saliva on macrophages is poorly understood. Recently, we showed that *L. longipalpis *saliva activates lipid body (LB) formation in resident macrophages committed with PGE_2_ production by COX-2 enzyme (Figure 3) [51]. Lipid bodies are intracellular sites related with eicosanoid production, and their formation can be triggered by activation via different intracellular pathways (as reviewed in [57]). In this context, *L. longipalpis *saliva activated ERK-1/2 and PKC phosphorylation and the inhibition of both pathways resulted in blockade of saliva-induced PGE_2_ production by macrophages [51]. PGE_2_ modulates the macrophage response during *Leishmania *infection in macrophages [58, 59] and is related with parasite dissemination after infection; however, the role of saliva in the PGE_2_ released by macrophages during *Leishmania *infection remains to be addressed. Further studies will be necessary to clarify the importance of eicosanoids stimulated by sand fly saliva in macrophage clearance of parasites and consequently in parasite transmission after sand fly bite.

## 6. Neutrophils and *L. longipalpis* Saliva: A Neglected Interaction on Scenery of *Leishmania* Infection

Looking to the neutrophils as a significant host-defense cell player in both innate and adaptive response of immune system, it is surprising that few works have attempted to investigate the consequences of vector's saliva and neutrophils interaction in the pathogenesis of leishmaniasis. The reasons to encourage this special attention rise from several lines of evidence showing that neutrophils participate in *Leishmania* immunopathogenesis, by uptaking promastigote forms, producing cytokines and inflammatory mediators or interacting with macrophages enhancing infection (as reviewed in [60, 61]).

Neutrophils are considered as an initial target of *Leishmania* infection [40, 62], and they are implicated in the immunopathogenesis of murine leishmaniasis [50, 63, 64]. Moreover, significant numbers of neutrophils are present at the inoculation site, lesions, and draining lymph nodes from *Leishmania*-infected mice [31, 63, 65–67]. In addition, *Leishmania* parasites undergo a silent entry into macrophages inside phagocytosed neutrophils, thus reinforcing the role of neutrophils on establishment of *Leishmania* infection [68]. *Leishmania donovani* inhibition of traffic into lysosome-derived compartments in short-lived neutrophils was suggested as a key process for the subsequent establishment of long-term parasitism [69]. On the other hand, neutrophils have also been implicated in parasite control. Phagocytosis of *L. major* by human neutrophils led to parasite killing [70]. Human neutrophils were capable to kill *L. donovani* by oxidative mechanisms [71], and, more recently, it was described the involvement of NET's (Neutrophil Extracellular Traps) on *L. amazonensis* destruction [72].

One elegant approach that reinforced the essential role for neutrophils in leishmaniasis revealed the presence of *Leishmania*-infected neutrophil on the sand fly bite site [40]. However, in that work, although the sustained neutrophil recruitment had been evident only in response to the sand fly bite, the authors did not attribute the neutrophil influx to vector salivary components. Surprisingly, besides neutrophil recruitment, there are no previous reports on further effects of sand fly saliva on neutrophil inflammatory response. Interestingly, studies performed with tick saliva disclose that the inhibition of neutrophil functions favors the initial survival of spirochetes [73–75].

Our group has recently shown the first evidence of direct effect of *L. longipalpis* salivary components on C57BL/6 mice neutrophils [76]. In summary, we described that saliva from *L. longipalpis* triggers apoptosis of inflammatory neutrophils obtained from C57BL/6 peritoneal cavity (Figure 3). The proapoptotic effect of saliva was due to caspase activation and FasL expression on neutrophil surface. Although salivary glands from blood feeding vectors have a variety of components [76], it seems that the proapoptosis compound in *L. longipalpis* saliva is a protein. However, further work is required to elucidate which protein or proteins act in this process. Additional helpful information from this study is that preincubation of *L. longipalpis* saliva with anti-saliva antibodies abrogated neutrophil apoptosis. This allows us to propose that proapoptotic component from *L. longipalpis* saliva could be target for the host's antibodies.

Moreover, neutrophil apoptosis induced by *L. longipalpis* saliva was also increased in the presence of *L. chagasi* [76]. This is particularly interesting by reinforcing the synergistic effect of both vector component and parasite on host inflammatory response, as have been observed in cell chemotaxis [42]. Interestingly, saliva from *L. longipalpis* enhanced *L. chagasi* viability inside neutrophils. This effect was attributed to modulation of neutrophil inflammatory response [76], as treatment of neutrophils with a pan caspase inhibitor (z-VAD) and a COX-2 inhibitor (NS-398) abrogated the increased parasite burden observed. Finally, we also described a novel inflammatory function of *L. longipalpis* saliva on neutrophils, stimulating MCP-1 production, able to attract macrophages *in vitro*. Even though chemotatic activity from *L. longipalpis *saliva has been previously reported, this is the first demonstration that saliva modifies directly the neutrophil inflammatory function, inducing the release of chemotatic factors by these cells.

## 7. Future Directions

In this paper, we explored the new inflammatory events induced by *L. longipalpis* in the recruitment and cellular function of leukocytes, as well as the repercussion to *L. chagasi *infection. The understanding of protective mechanisms regarding the initial steps of host's response to salivary molecules that can correlate with resistance or susceptibility to *Leishmania* has been poorly explored. Further investigation should address factors that determine the success of *Leishmania* infection. Identifying new escape mechanisms used by *Leishmania* associated to the pharmacological complexity of the sand fly saliva remains a challenge. In this scenario, phylogenetic implications between vector and *Leishmania* species can result in distinct action under host cells. The insights from the inflammatory scenery approached here, as lipid body induction in macrophages and apoptotic death of neutrophils, need to be investigated during the interaction between saliva from other sand fly and *Leishmania* species. Another important point is that these inflammatory effects were detected in salivary gland extract of sand fly vector. However, recombinants proteins from *L. longipalpis* saliva that presented known immunogenic role should be tested as inducers of these inflammatory events during infection by *Leishmania* sp. The studies discussed here suggest that saliva components can act on virulence factors from parasite surface in the first steps involved the recognition, resistance to oxidative mechanisms, and modulation of inflammatory mediators' produced by host cells. However, this finding seems to be part of a “large puzzle,” since they are viewed in isolation, by methodological limitations. Recent emerging imaging technologies have opened the possibility to monitor the process of* Leishmania*-host cell interaction in real time from the first moment upon sand fly bite, allowing understanding of molecular and cellular mechanisms in *Leishmania* experimental infection. These advances will enable future integrated studies that may increase understanding of immunopathogenic mechanisms induced by saliva in this intricate and fascinating interaction.

##  Conflict of Interests

The authors have no financial or other conflicts to declare.

## References

[1] Desjeux P (2010). Prevention of Leishmania donovani infection. *British Medical Journal*.

[2] Harhay MO, Olliaro PL, Vaillant M (2011). Who is a typical patient with visceral leishmaniasis? Characterizing the demographic and nutritional profile of patients in Brazil, East Africa, and South Asia. *American Journal of Tropical Medicine and Hygiene*.

[3] Kaye P, Scott P (2011). Leishmaniasis: complexity at the host-pathogen interface. *Nature Reviews Microbiology*.

[4] Ribeiro JMC (1995). Blood-feeding arthropods: live syringes or invertebrate pharmacologists?. *Infectious Agents and Disease*.

[5] Andrade BB, De Oliveira CI, Brodskyn CI, Barral A, Barral-Netto M (2007). Role of sand fly saliva in human and experimental leishmaniasis: current insights. *Scandinavian Journal of Immunology*.

[6] Ribeiro JMC, Francischetti IMB (2003). Role of arthropod saliva in blood feeding: sialome and post-sialome perspectives. *Annual Review of Entomology*.

[7] Lerner EA, Ribeiro JMC, Nelson RJ, Lerner MR (1991). Isolation of maxadilan, a potent vasodilatory peptide from the salivary glands of the sand fly lutzomyia longipalpis. *Journal of Biological Chemistry*.

[8] Lerner EA, Shoemaker CB (1992). Maxadilan: cloning and functional expression of the gene encoding this potent vasodilator peptide. *Journal of Biological Chemistry*.

[9] Guilpin VO, Swardson-Olver C, Nosbisch L, Titus RG (2002). Maxadilan, the vasodilator/immunomodulator from Lutzomyia longipalpis sand fly saliva, stimulates haematopoiesis in mice. *Parasite Immunology*.

[10] Bozza M, Soares MBP, Bozza PT (1998). The PACAP-type I receptor agonist maxadilan from sand fly saliva protects mice against lethal endotoxemia by a mechanism partially dependent on IL-10. *European Journal of Immunology*.

[11] Soares MBP, Titus RG, Shoemaker CB, David JR, Bozza M (1998). The vasoactive peptide maxadilan from sand fly saliva inhibits TNF-*α* and induces IL-6 by mouse macrophages through interaction with the pituitary adenylate cyclase-activating polypeptide (PACAP) receptor. *Journal of Immunology*.

[12] Charlab R, Valenzuela JG, Rowton ED, Ribeiro JMC (1999). Toward an understanding of the biochemical and pharmacological complexity of the saliva of a hematophagous sand fly Lutzomyia longipalpis. *Proceedings of the National Academy of Sciences of the United States of America*.

[13] Černá P, Mikeš L, Volf P (2002). Salivary gland hyaluronidase in various species of phlebotomine sand flies (Diptera: psychodidae). *Insect Biochemistry and Molecular Biology*.

[14] Ribeiro JMC, Spielman A (1986). Ixodes dammini: salivary anaphylatoxin inactivating activity. *Experimental Parasitology*.

[15] Anderson JM, Oliveira F, Kamhawi S (2006). Comparative salivary gland transcriptomics of sandfly vectors of visceral leishmaniasis. *BMC Genomics*.

[16] Valenzuela JG, Garfield M, Rowton ED, Pham VM (2004). Identification of the most abundant secreted proteins from the salivary glands of the sand fly Lutzomyia longipalpis, vector of Leishmania chagasi. *Journal of Experimental Biology*.

[17] Barral A, Honda E, Caldas A (2000). Human immune response to sand fly salivary gland antigens: a useful epidemiological marker?. *American Journal of Tropical Medicine and Hygiene*.

[18] Vinhas V, Andrade BB, Paes F (2007). Human anti-saliva immune response following experimental exposure to the visceral leishmaniasis vector, Lutzomyia longipalpis. *European Journal of Immunology*.

[19] Xu X, Oliveira F, Chang BW (2011). Structure and function of a "yellow" protein from saliva of the sand fly Lutzomyia longipalpis that confers protective immunity against Leishmania major infection. *Journal of Biological Chemistry*.

[20] Soares RPP, Turco SJ (2003). Lutzomyia longipalpis (Diptera: Psychodidae: Phlebotominae): a review. *Anais da Academia Brasileira de Ciencias*.

[21] Belkaid Y, Kamhawi S, Modi G (1998). Development of a natural model of cutaneous leishmaniasis: powerful effects of vector saliva and saliva preexposure on the long-term outcome of Leishmania major infection in the mouse ear dermis. *Journal of Experimental Medicine*.

[22] Kamhawi S (2000). The biological and immunomodulatory properties of sand fly saliva and its role in the establishment of Leishmania infections. *Microbes and Infection*.

[23] Silva F, Gomes R, Prates D (2005). Inflammatory cell infiltration and high antibody production in BALB/c mice caused by natural exposure to Lutzomyia longipalpis bites. *American Journal of Tropical Medicine and Hygiene*.

[24] Gomes RB, Brodskyn C, De Oliveira CI (2002). Seroconversion against Lutzomyia longipalpis saliva concurrent with the development of anti-Leishmania chagasi delayed-type hypersensitivity. *Journal of Infectious Diseases*.

[25] Aquino DM, Caldas AJ, Miranda JC, Silva AA, Barral-Netto M, Barral A (2010). Epidemiological study of the association between anti-Lutzomyia longipalpis saliva antibodies and development of delayed-type hypersensitivity to Leishmania antigen. *American Journal of Tropical Medicine and Hygiene*.

[26] Teixeira C, Gomes R, Collin N (2010). Discovery of markers of exposure specific to bites of Lutzomyia longipalpis, the vector of Leishmania infantum chagasiin Latin America. *PLoS Neglected Tropical Diseases*.

[27] Souza AP, Andrade BB, Aquino D (2010). Using recombinant proteins from Lutzomyia longipalpis saliva to estimate human vector exposure in visceral leishmaniasis endemic areas. *PLoS Neglected Tropical Diseases*.

[28] Costa DJ, Favali C, Clarêncio J (2004). Lutzomyia longipalpis salivary gland homogenate impairs cytokine production and costimulatory molecule expression on human monocytes and dendritic cells. *Infection and Immunity*.

[29] Kamhawi S, Belkaid Y, Modi G, Rowton E, Sacks D (2000). Protection against cutaneous leishmaniasis resulting from bites of uninfected sand flies. *Science*.

[30] Belkaid Y, Valenzuela JG, Kamhawi S, Rowton E, Sacks DL, Ribeiro JMC (2000). Delayed-type hypersensitivity to Phlebotomus papatasi sand fly bite: an adaptive response induced by the fly?. *Proceedings of the National Academy of Sciences of the United States of America*.

[31] De Moura TR, Novais FO, Oliveira F (2005). Toward a novel experimental model of infection to study American cutaneous leishmaniasis caused by Leishmania braziliensis. *Infection and Immunity*.

[32] Valenzuela JG, Belkaid Y, Garfield MK (2001). Toward a defined anti-Leishmania vaccine targeting vector antigens: characterization of a protective salivary protein. *Journal of Experimental Medicine*.

[33] Oliveira F, Lawyer PG, Kamhawi S, Valenzuela JG (2008). Immunity to distinct sand fly salivary proteins primes the anti-leishmania immune response towards protection or exacerbation of disease. *PLoS Neglected Tropical Diseases*.

[34] Gomes R, Teixeira C, Teixeira MJ (2008). Immunity to a salivary protein of a sand fly vector protects against the fatal outcome of visceral leishmaniasis in a hamster model. *Proceedings of the National Academy of Sciences of the United States of America*.

[35] Tavares NM, Silva RA, Costa DJ (2011). Lutzomyia longipalpis saliva or salivary protein LJM19 protects against Leishmania braziliensis and the saliva of its vector, Lutzomyia intermedia. *PLoS Neglected Tropical Diseases*.

[36] Collin N, Gomes R, Teixeira C (2009). Sand fly salivary proteins induce strong cellular immunity in a natural reservoir of visceral leishmaniasis with adverse consequences for Leishmania. *PLoS Pathogens*.

[37] Donnelly KB, Lima HC, Titus RG (1998). Histologic characterization of experimental cutaneous leishmaniasis in mice infected with Leishmania braziliensis in the presence or absence of sand fly vector salivary gland lysate. *Journal of Parasitology*.

[38] Paranhos M, dos Santos WC, Sherlock I, Oliveira GG, de Carvalho LC (1993). Development of eosinophilia in dogs intradermically inoculated with sand fly saliva and Leishmania (Leishmania) Chagasi stationary-phase promastigotes. *Memorias do Instituto Oswaldo Cruz*.

[39] Robinson DS, Kay AB, Wardlaw AJ (2002). Eosinophils. *Clinical allergy and immunology*.

[40] Peters NC, Egen JG, Secundino N (2008). In vivo imaging reveals an essential role for neutrophils in leishmaniasis transmitted by sand flies. *Science*.

[41] Zer R, Yaroslavski I, Rosen L, Warburg A (2001). Effect of sand fly saliva on Leishmania uptake by murine macrophages. *International Journal for Parasitology*.

[42] Teixeira CR, Teixeira MJ, Gomes RBB (2005). Saliva from Lutzomyia longipalpis induces CC chemokine ligand 2/monocyte chemoattractant protein-1 expression and macrophage recruitment. *Journal of Immunology*.

[43] Mirkovich AM, Galelli A, Allison AC, Modabber FZ (1986). Increased myelopoiesis during Leishmania major infection in mice: generation of “safe targets”, a possible way to evade the immune mechanism. *Clinical and Experimental Immunology*.

[44] Monteiro MC, Nogueira LG, Almeida Souza AA, Ribeiro JMC, Silva JS, Cunha FQ (2005). Effect of salivary gland extract of Leishmania vector, Lutzomyia longipalpis, on leukocyte migration in OVA-induced immune peritonitis. *European Journal of Immunology*.

[45] Monteiro MC, Lima HC, Souza AAA, Titus RG, Romão PRT, Cunha FDQ (2007). Effect of Lutzomyia longipalpis salivary gland extracts on leukocyte migration induced by Leishmania major. *American Journal of Tropical Medicine and Hygiene*.

[46] Nathan C (2006). Neutrophils and immunity: challenges and opportunities. *Nature Reviews Immunology*.

[47] Lawrence T, Willoughby DA, Gilroy DW (2002). Anti-inflammatory lipid mediators and insights into the resolution of inflammation. *Nature Reviews Immunology*.

[48] Savill JS, Henson PM, Haslett C (1989). Phagocytosis of aged human neutrophils by macrophages is mediated by a novel “charge-sensitive”recognition mechanism. *Journal of Clinical Investigation*.

[49] Afonso L, Borges VM, Cruz H (2008). Interactions with apoptotic but not with necrotic neutrophils increase parasite burden in human macrophages infected with Leishmania amazonensis. *Journal of Leukocyte Biology*.

[50] Ribeiro-Gomes FL, Otero AC, Gomes NA (2004). Macrophage interactions with neutrophils regulate leishmania major infection. *Journal of Immunology*.

[51] Araújo-Santos T, Prates DB, Andrade BB (2010). Lutzomyia longipalpis saliva triggers lipid body formation and prostaglandin E2 production in murine macrophages. *PLoS Neglected Tropical Diseases*.

[52] Hall LR, Titus RG (1995). Sand fly vector saliva selectively modulates macrophage functions that inhibit killing of Leishmania major and nitric oxide production. *Journal of Immunology*.

[53] Theodos CM, Titus RG (1993). Salivary gland material from the sand fly Lutzomyia longipalpis has an inhibitory effect on macrophage function in vitro. *Parasite Immunology*.

[54] Norsworthy NB, Sun J, Elnaiem D, Lanzaro G, Soong L (2004). Sand Fly Saliva Enhances Leishmania amazonensis Infection by Modulating Interleukin-10 Production. *Infection and Immunity*.

[55] Katz O, Waitumbi JN, Zer R, Warburg A (2000). Adenosine, AMP, and protein phosphatase activity in sandfly saliva. *American Journal of Tropical Medicine and Hygiene*.

[56] Brodie TM, Smith MC, Morris RV, Titus RG (2007). Immunomodulatory effects of the Lutzomyia longipalpis salivary gland protein maxadilan on mouse macrophages. *Infection and Immunity*.

[57] Bozza PT, Bakker-Abreu I, Navarro-Xavier RA, Bandeira-Melo C (2011). Lipid body function in eicosanoid synthesis: an update. *Prostaglandins Leukotrienes and Essential Fatty Acids*.

[58] Lonardoni MVC, Barbieri CL, Russo M, Jancar S (1994). Modulation of Leishmania (L.) amazonensis growth in cultured mouse macrophages by prostaglandins and platelet activating factor. *Mediators of Inflammation*.

[59] Pinheiro RO, Nunes MP, Pinheiro CS (2009). Induction of autophagy correlates with increased parasite load of Leishmania amazonensis in BALB/c but not C57BL/6 macrophages. *Microbes and Infection*.

[60] Charmoy M, Auderset F, Allenbach C, Tacchini-Cottier F (2010). The prominent role of neutrophils during the initial phase of infection by Leishmania parasites. *Journal of Biomedicine and Biotechnology*.

[61] Appelberg R (2007). Neutrophils and intracellular pathogens: beyond phagocytosis and killing. *Trends in Microbiology*.

[62] van Zandbergen G, Solbach W, Laskay T (2007). Apoptosis driven infection. *Autoimmunity*.

[63] Tacchini-Cottier F, Zweifel C, Belkaid Y (2000). An immunomodulatory function for neutrophils during the induction of a CD4+ TH2 response in BALB/c mice infected with Leishmania major. *Journal of Immunology*.

[64] Novais FO, Santiago RC, Báfica A (2009). Neutrophils and macrophages cooperate in host resistance against Leishmania braziliensis infection. *Journal of Immunology*.

[65] Barral-Netto M, De Freitas LAR, Andrade ZA (1987). Histopathologic changes induced by vaccination in experimental cutaneous leishmaniasis of BALB/c mice. *American Journal of Pathology*.

[66] Pompeu ML, Freitas LA, Santos MLV, Khouri M, Barral-Netto M (1991). Granulocytes in the inflammatory process of BALB/c mice infected by Leishmania amazonensis. A quantitative approach. *Acta Tropica*.

[67] Lima GMAC, Vallochi AL, Silva UR, Bevilacqua EMAF, Kiffer MMF, Abrahamsohn IA (1998). The role of polymorphonuclear leukocytes in the resistance to cutaneous Leishmaniasis. *Immunology Letters*.

[68] Van Zandbergen G, Klinger M, Mueller A (2004). Cutting edge: neutrophil granulocyte serves as a vector for Leishmania entry into macrophages. *Journal of Immunology*.

[69] Gueirard P, Laplante A, Rondeau C, Milon G, Desjardins M (2008). Trafficking of Leishmania donovani promastigotes in non-lytic compartments in neutrophils enables the subsequent transfer of parasites to macrophages. *Cellular Microbiology*.

[70] Laufs H, Müller K, Fleischer J (2002). Intracellular survival of Leishmania major in neutrophil granulocytes after uptake in the absence of heat-labile serum factors. *Infection and Immunity*.

[71] Pearson RD, Steigbigel RT (1981). Phagocytosis and killing of the protozoan Leishmania donovani by human polymorphonuclear leukocytes. *Journal of Immunology*.

[72] Guimarães-Costa AB, Nascimento MTC, Froment GS (2009). Leishmania amazonensis promastigotes induce and are killed by neutrophil extracellular traps. *Proceedings of the National Academy of Sciences of the United States of America*.

[73] Guo X, Booth CJ, Paley MA (2009). Inhibition of neutrophil function by two tick salivary proteins. *Infection and Immunity*.

[74] Montgomery RR, Lusitani D, De Boisfleury Chevance A, Malawista SE (2004). Tick saliva reduces adherence and area of human neutrophils. *Infection and Immunity*.

[75] Ribeiro JMC, Weis JJ, Telford SR (1990). Saliva of the tick Ixodes dammini inhibits neutrophil function. *Experimental Parasitology*.

[76] Prates DB, Araújo-Santos T, Luz NF (2011). *Lutzomyia longipalpis* saliva drives apoptosis and enhances parasite burden in neutrophils. *Journal of Leukocyte Biology*.

